# Mental health preparedness and response to epidemics focusing on COVID-19 pandemic: a qualitative study in Iran

**DOI:** 10.1186/s12889-024-19526-2

**Published:** 2024-07-24

**Authors:** Khadijeh Akbari, Armin Zareiyan, Arezoo Yari, Mehdi Najafi, Maryam Azizi, Abbas Ostadtaghizadeh

**Affiliations:** 1 Department of Health in Emergencies and Disasters, School of Public Health, Tehran University of Medical Sciences, Poorsina Ave, Tehran, 14177-43578 Iran; 2 Department of Nursing, 501 Hospital (Imam Reza), Aja University of Medical Sciences, Tehran, Iran; 3 Department of Health in Emergencies and Disasters, School of Nursing, Aja University of Medical Sciences, Tehran, Iran; 4 Social Determinants of Health Research Center, Research Institute for Health Development, Kurdistan University of Medical Sciences, Sanandaj, Iran; 5 Department of Health in Emergencies and Disasters, School of Medicine, Kurdistan University of Medical Sciences, Sanandaj, Iran; 6 Department of Rescue and Relief, Iran Helal Applied Science Higher Education Institute, Tehran, Iran; 7 Department of Health in Disasters and Emergencies, School of Nursing, Aja University of Medical Sciences, Tehran, Iran

**Keywords:** Mental health, Epidemics, COVID-19, Health system, Iran

## Abstract

**Background:**

During epidemics, the number of individuals whose mental health is affected is greater than those affected by the infection itself. This is because psychological factors have a direct relationship with the primary causes of the disease and mortality worldwide. Therefore, an increasing investment in research and strategic actions for mental health is essential globally, given the prevalence of infectious diseases.

The aim of this study was to elucidate and describe the strategies for mental health preparedness and response during epidemics, with a focus on the COVID-19 pandemic in Iran.

**Methods:**

A qualitative study was conducted in Iran from 2022 to 2023. Purposeful Sampling was employed, continuing until data saturation was achieved. Data collection involved semi-structured interviews and observational notes with 20 managers and experts possessing expertise, experience, and knowledge in mental health. Ultimately, the participants' opinions, based on their experiences, were analyzed using the qualitative content analysis method with a conventional approach, resulting in the categorization of data into codes, subcategories, and categories.

**Results:**

The study revealed participants' opinions and experiences, categorized into two overarching categories: Preparedness, Policy-Making, and Planning Strategies (with four subcategories), and Response Strategies (comprising thirteen subcategories).

**Conclusion:**

The opinions and experiences of managers and experts in this study revealed that an appropriate mental health response during pandemics requires preparedness before the occurrence of such crises and the implementation of suitable response strategies after the occurrence. Managers, policymakers, and decision-makers in this field should pay attention to the solutions derived from the experiences of such crises to respond more preparedly in the future.

**Supplementary Information:**

The online version contains supplementary material available at 10.1186/s12889-024-19526-2.

## Background

Mental health is an essential component of human well-being that can be significantly impacted during pandemics and epidemics [1]. Communicable diseases, especially those requiring isolation and quarantine, pose substantial risks to individuals' mental health. Anxiety, stress, depression, and grief are common mental health issues experienced during and after the outbreak of a disease [2]. During the Ebola virus epidemic in West Africa during 2014–2016, significant mental health challenges were reported among the affected population [3]. The recent emergence of the COVID-19 pandemic has created conditions that have exacerbated many factors that can weaken mental health. Before 2020, mental disorders were among the leading contributors to the global burden of disease, with depression and anxiety disorders being the primary factors [4]. During the first year of the COVID-19 pandemic, the global prevalence of these disorders increased by 25% [5]. As observed in previous studies on other severe epidemics, the overall impact of a pandemic on mental health is not transient and is likely to persist for a long time even after the pandemic has ended [6]. Unpredictability [7], lack of preparedness, inconsistencies in guidelines, quarantines, containment strategies, unemployment, financial losses, physical distancing, isolation, chaos, uncertainty [8], ease of access to communication strategies and transmission of sensational misinformation and disinformation [9] are among the factors that lead to increased emotional distress, anxiety, and depression [8].

While health emergencies have been a recurring aspect of human history, the global community found itself unprepared for the impact of the COVID-19 pandemic [7, 10]. According to a World Health Organization survey, the pandemic disrupted or halted mental health services in 93% of the world's countries, coinciding with a rising demand for mental health support [11]. The longstanding prevalence of mental illnesses has posed a persistent public health challenge. In the United States, the provision of high-quality healthcare services faces formidable challenges attributed to various gaps in the mental health care system. These gaps include disparities in treatment, elevated drug prices, fragmented systems, ineffective policies, structural issues, workforce shortages, limited access, and financial barriers. The urgency to address these gaps has intensified, particularly in light of the escalating mental health issues stemming from the COVID-19 pandemic [8].

The sudden outbreak of public health crises always presents significant challenges for the mental health care system. Effective management of communicable disease pandemics such as COVID-19 requires approaches that encompass various aspects affecting outcomes. Timely provision of mental health care during epidemics is crucial, with interventions tailored to different stages of the epidemic, including during and after the outbreak [12]. Policy decisions should prioritize reinforcing community-based care, providing support, enhancing capacity for public mental health research, ensuring easy access to healthcare services [8], and effectively supporting healthcare providers.As highlighted in Irandoost et al. [13], understanding the experiences, challenges, and adaptation strategies of healthcare providers is essential for improving mental health response during such crises [13].

The Islamic Republic of Iran has accumulated significant experience in providing social and psychological support during disasters over the past two decades. For example, in response to the inevitable mental health consequences of the COVID-19 pandemic, various measures were implemented nationwide. At the onset of the virus outbreak, the predominant feelings among the population were anxiety, worry, and confusion. The Ministry of Health and Medical Education (MOH) initiated several coping strategies to address stress and tension in society. These strategies included training healthcare workers in primary healthcare systems and establishing a helpline to increase access to care. Collaboration with other governmental sectors, such as the Islamic Republic of Iran Broadcasting (IRIB) and social media networks, was employed to ensure the efficient dissemination of information. Lessons learned from managing mental health issues during emergencies, particularly amid the COVID-19 pandemic, emphasize the importance of adopting a dynamic approach to address community mental health needs effectively [14].

Public mental health efforts aim to enhance disaster response [7] and integrate mental health interventions into global preparedness and response programs [15], informed by scientific evidence and cultural considerations. Further research is required to develop psychological interventions for improving mental health outcomes [16]. Qualitative studies, by exploring perspectives and experiences, facilitate a deeper understanding of phenomena. Our qualitative approach captured nuanced experiences and response strategies during the epidemics, providing insights not easily uncovered through quantitative methods. This study highlights the challenges and response strategies during the COVID-19 pandemic in Iran. The insights gained can guide future policies and interventions for similar crises. The researchers' experience in mental health and disaster response emphasizes the importance of integrating mental health support into preparedness plans and adopting a dynamic approach to community mental health needs during and after epidemics. The aim of this study was to elucidate and describe the strategies for mental health preparedness and response to epidemics focusing on the COVID-19 pandemic in Iran.

## Materials and methods

A qualitative study was conducted employing the content analysis method to elucidate and describe strategies and programs related to mental health preparedness and response to epidemics, with a focus on the COVID-19 pandemic in Iran during 2022–2023. Content analysis is a systematic method aimed at achieving depth and breadth in describing phenomena, leading to valid interpretations of information and the generation of new insights. It is particularly suitable for exploring individuals' experiences and perspectives on specific topics. In this study, conventional content analysis was used, wherein categories are derived concurrently with the analysis of interview text, allowing researchers to gain a better understanding of the phenomenon under study [17].

### Participants and setting

To ensure the maxim of variation, participant selection was purposeful, aiming to include individuals with diverse experiences and expertise relevant to the primary phenomenon or key concepts under investigation. The study encompassed managers, experts, and individuals with experience in mental health during epidemics and the COVID-19 pandemic, representing various academic and executive environments within responsible organizations. These organizations included the Ministry of Health, Social Welfare Organization, Psychiatric and Psychological Scientific Associations, municipalities, Iranian Red Crescent Society, National Disaster Management Organization, universities, and private sector entities. The exclusion criterion of unwillingness to participate helped maintain the integrity of the sample.

Coordination with potential participants was established through telephone communication, and their inclusion in the study was contingent on their willingness to participate. Selection criteria were based on knowledge, expertise, experience, and organizational affiliations, ensuring participants played active roles in policymaking, decision-making, service delivery, implementation, monitoring, and supervision in the mental health field. Additionally, input from academics was sought due to their valuable insights derived from studies in this field. Data collection continued until theoretical data saturation was achieved, indicating that no further data could be obtained. Deliberate selection of participants with varying opinions ensured diversity in the sample.

To ensure transparency and mitigate potential conflicts of interest during the interviews, participants were explicitly informed that their responses would be treated confidentially and would not impact their professional roles or affiliations. Furthermore, efforts were made to maintain impartiality throughout the interview process, emphasizing that their input would solely contribute to research findings and not influence their work. Informed consent was obtained from all participants as part of the research process. Privacy of information (including names, interview recordings, and transcripts) was strictly maintained, and coding was used instead of names to ensure confidentiality. Participants had the right to withdraw from the study at any time, and the option to share the results upon request was provided to them.

### Data collection

To collect the data, coordination and pre-scheduled appointments were made either in the workplace of the participants or virtually, and in-depth individual interviews were conducted with them. The semi-structured interviews were conducted using an interview guide. Initially, two unstructured interviews were conducted to determine the main interview outline and complete the interview guide questions. All interviews were conducted individually to ensure that participants could freely share their experiences and perspectives without influence from others.

At the beginning of the interviews, participants were provided with an explanation of the study's objectives. The interviews commenced in a friendly environment with several general questions, such as introducing themselves. They were then asked if they had experienced responding to mental health issues during the COVID-19 pandemic. If a participant had relevant experience, they were requested to describe it. Subsequently, the interviews continued with the following main questions:

How would you describe your positive and negative experiences in this field? In order to provide mental health response strategies, what issues and challenges have you encountered? Please describe them. What solutions do you employ to deal with these challenges? Please give an example. Based on your experiences during the COVID-19 pandemic, describe the actions that the healthcare system should take in response to mental health issues during epidemics.

All interviews were audio-recorded with permission. Furthermore, the researcher (KH.A.) took notes during the interviews to gather the data more comprehensively. Data analysis was conducted simultaneously with data collection.

### Data analysis

The data were analyzed using the qualitative content analysis method proposed by Graneheim and Lundman [18]. At the end of each interview session, in the first phase, the recorded interviews were listened to several times, and transcripts were created, incorporating verbatim accounts. Prior to analysis, in the second phase, the transcripts were read multiple times to familiarize the researchers (KH.A. and A.Y.) with the interviews. In the third phase, to the analyze the interviews, the transcripts were broken down into the smallest meaningful units and codes. These initial codes were then compared with each other, and similar codes were categorized into subcategories. Furthermore, by continually comparing the subcategories and based on their relevance and similarity, these subcategories were placed within the main categories, which contained the main themes and were somewhat abstract. To confirm the codes, the text was read multiple times (KH.A. and A.Y.). A third researcher (A.O.T.), with higher academic and executive expertise, refined the codes and categories in the final stage. Content analysis was performed on the data written in the Persian language before translation manually.

### Trustworthiness

The trustworthiness of a qualitative research study relies on the rigor of the methodology [18]. Four criteria for evaluating qualitative research are credibility, transferability, dependability, and confirmability [19].

For credibility, the researcher ensured trustworthiness and acceptability of the data through long-term and continuous involvement with the environment and participants, allocating an average duration of 10 months to data collection and analysis. All interview transcripts and analysis stages were reviewed by two expert individuals experienced in qualitative research, who provided initial coding. A third researcher refined the codes and categories in the final stage, incorporating supplementary comments. Additionally, interview transcripts were returned to some participants for feedback, with corrections made accordingly.

"Dependability" refers to the stability and reliability of data over time and under similar conditions. To assess dependability, an external audit was conducted by an individual with expertise in the field. Specifically, an external auditor with a PhD in health psychology, familiar with disaster-related research, reviewed the data to ensure consistency and reliability in its interpretation.

"Transferability" in qualitative research refers to how the findings of a study can be applied to other contexts or populations. To enhance transferability in this research, the researcher employed various strategies, including simultaneous data collection and analysis, ensuring coherence between research questions and methods, comparing results with other studies, providing a step-by-step report of each research stage, and involving a diverse spectrum of participants. This approach ensures that the findings are relevant to a broader audience beyond the specific study environment.

"Confirmability," which relates to the accuracy of all stages of research and the transparency of the research method, signifies the need to ensure that the findings are derived from the data and not influenced by the researcher's biases or preconceptions. In this study, rigorous measures were taken to maintain the accuracy and transparency of all research stages. Detailed documentation was conducted, encompassing the processes of data collection and analysis, as well as the researcher's notes and interpretations. This meticulous record-keeping aimed to provide interested readers with the ability to align the study with their own contexts and utilize it effectively.

## Results

In this study, a total of 20 participants took part, comprising 8 females (40%) and 12 males (60%). The mean age of the participants was 48.5 years, ranging from 35 to 64 years. Educational backgrounds varied, with one participant holding a master's degree and one possessing a postdoctoral degree. The remaining participants were medical specialists with doctoral degrees (Table 1). Due to geographical distances, interviews with three participants were conducted virtually. The interviews lasted 45 to 60 min, with an average duration of 52 min. All interviews were conducted in Persian. The study was conducted between August 2021 and June 2022.

**Table 1:** Demographic characteristics of participants in qualitative study

Demographic characteristics	Number (%)
**Gender**	
Female	8(40)
Male	12(60)
**Education level**	
Master’s degree	1(5)
Specialized doctorate degree	18(90)
Postdoctoral degree	1(5)
**Specialty**	
Health psychology	4(20)
Clinical psychology	4(20)
Psychiatry	3(15)
Psychiatric nursing	1(5)
Health in disasters and emergencies	5(25)
Infectious diseases	2(10)
Emergency medicine	1(5)
**Participants' organizational positions**	
Ministry of Health	3(15)
Social Welfare Organization	2(10)
Psychiatric and Psychological Scientific Associations	3(15)
Municipalities	2(10)
Iranian Red Crescent Society	2(10)
National Disaster Management Organization	1(5)
Universities	4(20)
Private sector and others	3(15)
**Years of work experience in the field of mental health**	
14(70)Less than 5	2(10)
5–15	4(20)
More than 15	14(70)

By analyzing the participants' interviews, their experiences were categorized into two main categories and 17 sub categories (Table 2).

**Table 2:** Results of the analysis of participants' data regarding mental health preparedness and response strategies during epidemics/pandemics

Category	Subcategory	Some codes
**Policy-making, planning, and preparedness**	**Mental health governance**	Leadership in mental health, collaboration of the Ministry of Health with other relevant authorities and institutions, perception of mental health risk in health organizations, prioritization of mental health in major health decisions
	**Policy-making and laws**	Formulation of transparent policies, improvement of laws, reevaluation in the policy domain, development of evidence-based strategies and policies reflecting the societal context, coherence in legislation, evidence-based policymaking, and persuading and sensitizing policymakers
	**Planning**	Planning for mental health response, formulating a comprehensive stress response program, developing plans tailored to the capacities and infrastructures of society, and creating programs for improving mental health issues
	**Training and exercise**	Training and development of professional and semi-professional groups, inclusion of specialized courses in relevant academic fields, designing educational programs to enhance mental health literacy among all healthcare system personnel, scenario development, implementation of scenario-based operational exercises, evaluation of exercises and continuous improvement
**Response strategies**	**Command and leadership**	Coordinated and integrated management, unified management and avoidance of parallel work, power and authority of command, deployment of an incident command system
	**Human resources**	Mobilizing a sufficient and well-trained human workforce, organizing volunteer forces, assisting relief workers, addressing the needs of response forces, having creative and efficient managers, and utilizing elites and experts
	**Financial resources**	Adequate financial resource provision and increasing the mental health budget during epidemics, targeted and equitable allocation of resources, financial support and incentives as effective strategies in response, expansion of insurance coverage in the field of mental health, support for the private sector in the mental health sector
	**Infrastructures**	Strengthening and upgrading current infrastructures, enhancing and developing Primary Health Care (PHC), strengthening mental health services in PHC, attention to the capacity of non-governmental organizations (NGOs), establishing a public response telephone system (such as the 4030 system in Iran) and social emergency (123)" in Iran
	**Monitoring and Research**	Establishing a central monitoring center, continuous mental health monitoring, implementing and strengthening the mental health monitoring system, activation of mental health care surveillance programs at centers, developing structures for guidance, control, evaluation, and social responsiveness, formation of electronic mental health records in electronic health systems in Iran (such as the SIB system in Iran), monitoring at entry points (university entry, military service, and employment processes, etc.), research on interventions and their effectiveness, facilitating research conduct, interdisciplinary research, utilizing experiences and lessons learned
	**Collaboration and coordination**	Engaging government and private sectors, soliciting participation and collaboration from other organizations and institutions, and inter sectoral collaboration and coordination
	**Identification of vulnerable groups**	Rapid and accurate identification of affected groups, attention to vulnerable and special groups (e.g., elderly, children, refugees, people with disabilities), focus on groups at risk of psychological harms (e.g., healthcare workers, frontline workers, individuals with pre-existing mental health conditions)
	**Provision of mental health services**	Early identification and screening of mental disorders, increased access to services, timely service delivery, provision of initial psychological assistance, delivery of standard services, equitable health service provision, providing safe and affordable services, adherence to ethical and privacy standards in service delivery, continuity of service provision, offering services tailored to the needs of recipients, development of new intervention models, provision of services based on primary, secondary, and tertiary prevention, use of free centers, and psychological rehabilitation through long-term interventions
	**Communication and information management**	Introduction of scientific references, introducing reliable information sources, providing accurate and timely information, Risk communication, combating disinformation, monitoring media and outlets, countering false information dissemination, establishing and consolidating coherent information databases, creating a unified electronic information system, formulating mechanisms for data access, developing powerful information systems, awareness-raising for distinguishing information sources
	**Public education and cultural promotion**	Providing empowerment training packages, offering psychological training, self-awareness, problem-solving, and emotional management training, resilience enhancement training, health and infection control training, education for all segments of society, needs-based education, understandable and user-friendly educational content, accessibility of educational materials, collaboration of various organizations in education, cultural promotion of mental health service utilization, addressing the stigma of mental illnesses, attention to social marketing in education and cultural promotion, promoting mental health products in cities
	**Employment of technology, tools, and tele-psychiatry**	Strengthening telemedicine, utilizing technology in psychoeducation, providing remote psychological interventions using the Internet and smartphones, using hotlines for social and mental emergencies, establishing support groups through technology, substituting and integrating therapy and counseling programs using technology, tailoring technology based on audience needs, ensuring technology accessibility, using technology to reduce costs, and developing technological infrastructure
	**Social support**	Attention to the collective fate among the general population, fostering hope for the future, promoting societal justice, combating individualistic thinking, filling the leisure time of families during quarantines, creating joyful and educational programs, developing entertainment suitable for different demographic groups, promoting sincere behavior, building social trust, establishing a public presence in management structures, maintaining continuous and dynamic communication with the public, encouraging public participation, and creating centers and associations
	**Economic support**	Support for employers, distribution of livelihood packages, assistance for unemployed individuals, financial support for those affected

### Policy-making, planning, and preparedness

This category encompassed all the actions that need to be taken in advance to reduce damage and ensure necessary preparedness in the field of mental health during epidemics. This main category composes four subcategories as follows:

### Mental health governance

The participants identified the governance of mental health as one of the effective strategies for an appropriate response. With mental health governance, the goals of the organization and the appropriate structure for achieving those goals, along with relevant laws and regulations, are established. Mental health should govern all levels and dimensions of the health-related organizations, and the impact of psychological factors on individual and societal health should be recognized as a priority. For example, participant number 5, a health psychologist, stated:

*"Absolutely, prioritizing mental health in health policies and programs is crucial for overall well-being. Raising awareness about the risks of neglecting mental health among relevant authorities is essential. Collaboration between the Ministry of Health and other agencies, along with the utilization of economic, social, and other resources, can help in effectively governing mental health. It's important to work together to ensure the mental well-being of individuals in our society."*

By incorporating these insights, organizations can develop comprehensive strategies that not only address immediate mental health needs but also build resilience against future epidemics. This collaborative approach ensures that mental health remains a central focus in public health planning and response efforts.

### Policy-making and laws

Developing transparent policies and laws for mental health during epidemics is considered a strategy for reducing damage and preparing for an appropriate response. The participants emphasized the need for clear policies. Participant number 11, a clinical psychologist, stated: *"There should be transparent policies in the field of mental health during health crises, and this issue should be promoted in all health policies of the country, and clear laws should be formulated."*

These policies should outline specific protocols and responsibilities for various stakeholders, ensuring a coordinated and efficient response. By having clear, established laws, mental health interventions can be systematically implemented, and resources can be allocated more effectively, ultimately enhancing the overall resilience of the healthcare system during epidemics.

### Planning

The participants emphasized the importance of planning for an appropriate response during the preparedness phase as one of the crucial elements. Participant number 4, a specialist in health in disasters and emergencies, expressed the following:

*"A comprehensive response plan should be considered before the outbreak of communicable diseases. Given the limitations of resources, planning should be done with a full understanding of capacities and infrastructure. Preparedness actions should be taken to provide an appropriate response during epidemics."*

Proper planning ensures that mental health services are seamlessly integrated into the overall health response, addressing both immediate and long-term needs. This proactive approach helps mitigate the impact of the epidemic on mental health, providing structured and timely support to affected individuals and communities.

### Training and exercise

Training and exercising preparedness programs before the occurrence of major epidemics are essential for an appropriate response, and they were among the proposed solutions by the participants. Participant number 15, a Psychiatrist, stated:

*"Training of personnel and conducting exercises for mental health programs before the outbreak of communicable diseases is necessary so that all responsive organizations can function effectively. Training and practical exercises should be updated to maintain a high level of preparedness for personnel and organizations."*

By engaging in continuous training and exercises, organizations can ensure that their teams are well-prepared to handle the psychological impacts of epidemics, providing timely and effective support to those in need. This proactive approach helps build resilience and ensures that mental health responses are integrated seamlessly into the broader epidemic response efforts.

### Response strategies

This category represented the solutions provided by the participants for an appropriate mental health response during epidemics, including:

### Command and leadership

Participants expressed the essentials of commanding in responding to emergency situations. The presence of unified leadership is crucial for integrated management, providing a framework for various organizations to work effectively together and synchronize their actions. Participant number 1, a specialist in health in disasters and emergencies, expressed:

*"Unified command and leadership are necessary during the response phase. The Ministry of Health should be the leading authority for mental health in our country, and all collaborating organizations should operate under its leadership, ensuring sufficient authority. The incident command structure should be activated during the response phase to ensure coordination among all organizations."*

Effective command and leadership not only streamline the decision-making process but also ensure that all organizations involved in the response are aligned in their efforts, thereby enhancing the overall effectiveness of the mental health response during epidemics.

### Human resources

The participants identified the human resources management and organization as the most critical principle for an appropriate response. They emphasized the importance of supporting responders during the response phase. Participant number 6, a clinical psychologist, stated: *"Healthcare workers have been under difficult conditions, with heavy and continuous work shifts, restrictions on interactions with family and friends, and concerns about contracting and transmitting the virus, causing them to endure significant pressure. Taking measures to ensure an adequate workforce for service delivery, appropriate work shifts, and providing equipment to maintain the safety of employees are crucial. Managers should attend to the various physical and psychological needs of responders."*

Ensuring the wellbeing of healthcare workers not only helps in maintaining their efficiency but also prevents burnout, thereby improving the overall response to mental health challenges during epidemics.

### Financial resources

Provision and organization of financial resources were highlighted as essential elements of the response strategies, emphasizing the need for provision of financial resources and their equitable distribution. Participant number 20, a specialist in health in disasters and emergencies, stated:

*"In the realm of mental health, given its costliness, there is a need for financial provision and investment. To achieve maximum efficiency with minimal harm, there must be equity in allocating both large and small-scale resources."* Moreover, Participant number 10, as a health psychologist, said:

*"It is essential to take seriously the provision of insurance coverage for mental health services in the country, ensuring that it leads to a reduced financial burden on the public. Additionally, supporting the private sector, which provides mental health services, and addressing their concerns are also crucial."* Ensuring that financial resources are adequately provided and equitably distributed is vital for maintaining the continuity and quality of mental health services, especially during crises.

### Infrastructures

The participants emphasized the need to strengthen and enhance the existing infrastructure of community mental health for the provision of mental health services during the response phase. Participant number 2, a psychiatric nurse, expressed:

*"Increasing capacity is crucial during epidemic response. Non-governmental organizations (NGOs) possess significant capacities at the grassroots level. These capacities can be utilized for educating the public and leveraging their capabilities and resources in times of response."*

Moreover, Participant number 6, a clinical psychiatric, said: *"It is essential to utilize and strengthen the existing infrastructure for delivering the intended services, and there is no need to create new structures. For instance, considering the valuable presence of the Primary Health Care (PHC) structure in our country, it is necessary to strengthen mental health services in primary healthcare."*

Utilizing and enhancing existing infrastructures ensures a more efficient and coordinated response to mental health needs during epidemics, leveraging already established systems and resources. This approach maximizes the effectiveness of response efforts by utilizing the capacities already present within organizations and communities.

### Monitoring and research

The participants emphasized the significance of both conducting research and monitoring mental health across all phases—pre-epidemic, epidemic, and post-epidemic—as crucial response strategies, including the establishment of centralized monitoring centers and the utilization of electronic health records. These measures can facilitate real-time monitoring of mental health indicators and trends, allowing for timely interventions and adjustments in response strategies. Participant number 13, a Psychiatrist, highlighted the necessity of robust mental health surveillance systems, stressing the importance of effective monitoring mechanisms. He stated:

*"It is necessary to strengthen mental health surveillance systems for various societal groups by the Ministry of Health. These systems should include robust mechanisms for monitoring mental health indicators and trends, such as electronic health records and centralized monitoring centers. With an awareness of the mental health status of individuals in society and the factors influencing it, more informed decisions can be made for mental health responses during epidemics. Additionally, the evaluation of implemented programs and the ability to make adjustments if necessary are essential components of effective monitoring."* Furthermore, Participant number 14, an emergency medicine specialist, stated:

*"Epidemics have various effects on the mental health of the population. Extensive research should be conducted in this area, focusing on effective interventions for the mental health of the population and their effectiveness… Existing knowledge should be transferred and applied."*

Investing in robust monitoring and research frameworks ensures ongoing assessment and adaptation of mental health responses across different phases of epidemics. By leveraging centralized monitoring centers and electronic health records, timely interventions can be implemented, addressing emerging mental health trends effectively.

### Collaboration and coordination

The responsible participation of all sectors and coordinated utilization of all capacities in mental health response during epidemics were highlighted as crucial strategies by the participants. Participant number 3, a health psychologist, expressed:

*"All sectors involved in mental health response, including the government, supporting groups, NGOs, Farzanegan Foundation, retirement homes, etc., should engage and participate. However, this participation of various organizations should not lead to conflicts. Coordination needs to be strengthened at a macro level, and contradictions in decision-making should be resolved. Sometimes, different decisions were made in managing the COVID-19 pandemic in our country, causing confusion and concern among the people."*

Ensuring effective collaboration and coordination among diverse sectors is crucial to avoid conflicts and enhance decision-making coherence during epidemic responses.

### Identification of vulnerable groups and individuals

The participants believed that one of the most important actions is to pay attention to vulnerable groups. For example, participant number 17, a specialist in health in disasters and emergencies, stated:

*"Resources and necessary infrastructure should be considered to support vulnerable groups. Identifying and tracing vulnerable and at-risk groups for service provision and follow-up is crucial. We should strive to provide automated services to these segments. For example, we currently have a significant number of bereaved families who have lost their loved ones. Do we have any plans in place for them?"*

Ensuring targeted support and services for vulnerable populations can enhance epidemic response effectiveness*.*

### Mental health services

The participants emphasized that providing mental health services is one of the most crucial actions in the response phase. They underscored the necessity for delivering services that are safe, affordable, and timely. Participant number 7, a psychiatrist, stated:

*"In the response phase, mental health services should be upgraded and expanded in terms of personnel, equipment, and space to increase public access to mental health services. Initial psychological aid should be provided, and services must be tailored to meet specific needs. New intervention models should be developed, and spaces for simply listening to people's concerns should be established. Free counseling centers within the community should be expanded. The privacy of individuals should be preserved and a sense of security should be ensured in receiving services*." Ensuring the affordability and accessibility of mental health services is crucial to meet the diverse needs of the population during epidemics.

### Communication and information management

Timely and accurate communication and information management are the key solutions that contribute to maintaining mental health. Participant number 9, a specialist in health in disasters and emergencies, stated:

*"Providing accurate and timely information is crucial during epidemics for the mental health of the community. Offering timely and accurate information and raising awareness among the public helps reduce the psychological impact of receiving various rumors. During an epidemic, combating misinformation is also vital, and it requires proper and effective communication. For example, we observe that misinformation leads to public confusion and prompts deviation from health guidelines, such as wearing masks or seeking appropriate treatments. At times, vaccine hesitancy due to misinformation has been evident."* Furthermore, Participant number 19, an infectious disease specialist, said:

*"A unified electronic mental health information system aids in mental health management. There should be a mechanism for accessing the required data, and the information from mental health records at health centers should be integrated and consolidated. Accurate data and statistics assist us in providing better responses."* Ensuring secure and efficient communication channels during epidemics is essential for disseminating accurate information and countering misinformation effectively.

### Public education and cultural promotion

Participants considered education and cultural initiatives as essential for preserving and enhancing mental health during the response phase. Participant number 8,* a Clinical Psychologist, stated:*

*"One important solution is to provide empowerment educational packages to the public. Psychological training should be given to teach people how to take care of themselves. Providing education on lifestyle skills, self-care, self-awareness-based training, problem-solving, and emotional management to the general public is beneficial. Essentially, people should be informed about what has happened, what reactions exist, what the future holds, and where to access services."*

Education and cultural promotion play a crucial role in fostering resilience and understanding among the population during epidemics, promoting proactive mental health practices and reducing stigma associated with seeking help.

### Employment of technology, tools, and tele-psychiatry

The utilization of technology in various dimensions of mental health during the response phase to epidemics of communicable diseases was identified as highly practical and essential. Participant number 12,* a health Psychologist*, stated:

*"The use of technology in the field of mental health during the COVID-19 pandemic was strongly emphasized, and it is necessary to strengthen telemedicine in the field of mental health (screening, treatment, etc.). Remote psychological interventions using the Internet and smart phones will help us to provide better responses in the future. For example, by utilizing these technologies, we can create support groups, facilitate discussions, and provide an outlet for individuals to express their feelings."*

Leveraging technology, including telemedicine and digital platforms, enhances accessibility to mental health services, supports remote psychological interventions, and fosters community engagement and support during epidemics.

### Social support

Paying attention to social support and gaining the trust and participation of the public were mentioned as effective response strategies. Participant number 16, a Clinical Psychologist, stated:

*"Responsible engagement of the public significantly contributes to improving the mental health of the society during epidemics. It is essential to strengthen the spirit of social solidarity and, through sincere interactions, build their trust."* Fostering social support networks and promoting community solidarity are crucial for enhancing mental well-being and resilience during epidemics.

### Economic support

Financial and economic support for individuals affected by pandemics was also among the proposed solutions by the participants. Participant number 18, a Clinical Psychologist, stated:

*"Supporting individuals who lose their jobs or face economic hardships due to pandemics should be a priority, as job and financial insecurities can have negative impacts on mental health."*

Ensuring adequate economic support for affected individuals is essential for mitigating the psychological impact of economic uncertainties during pandemics.

## Discussion

**Fig. 1:**
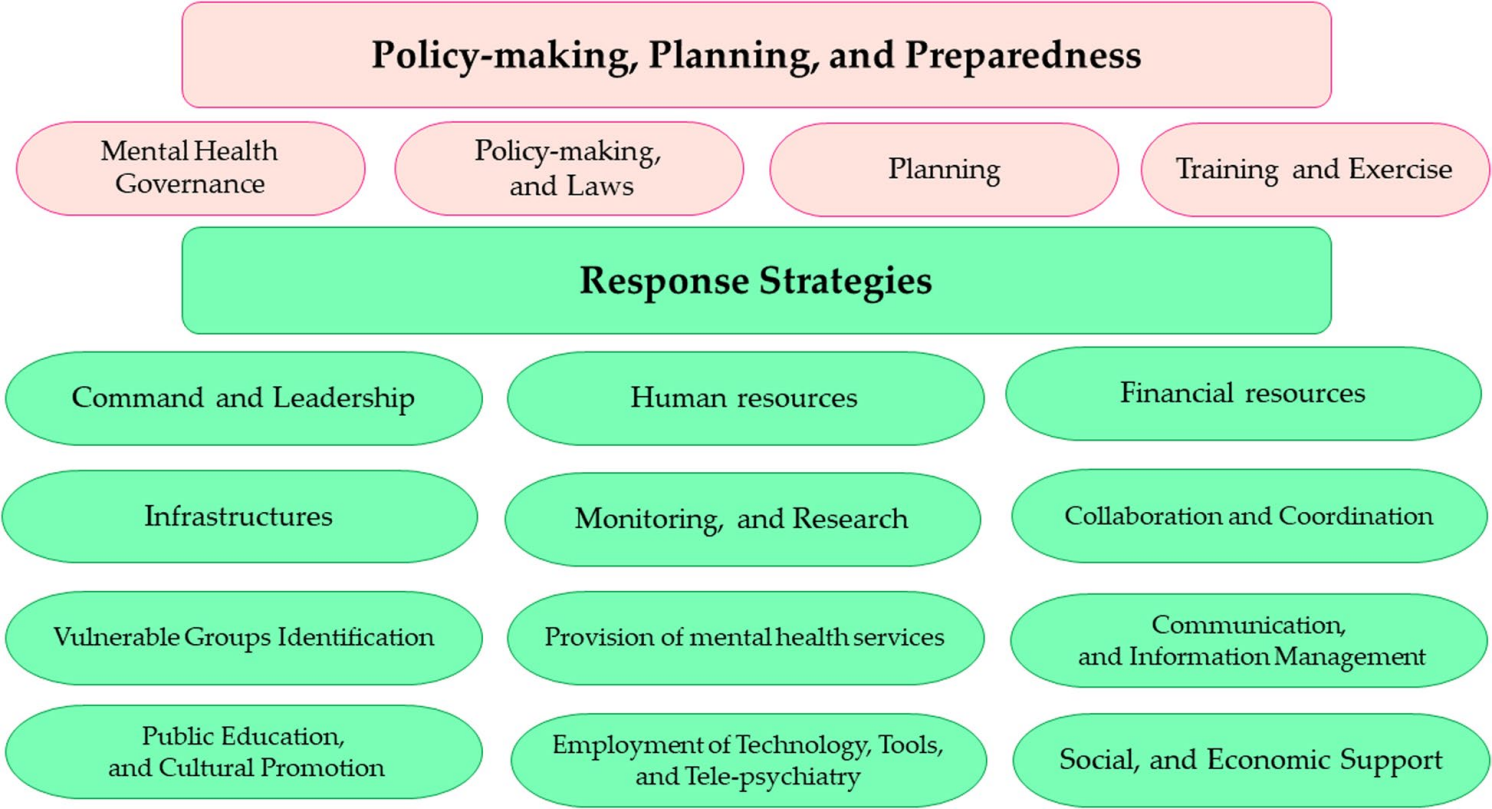
Participants' experiences of key strategies for mental health preparedness and response during epidemics

Numerous solutions exist for mental health preparedness and response during pandemics. However, among studies related to this topic, a few have provided a comprehensive examination of these solutions. Through content analysis of the results of this study, a relatively comprehensive set of effective strategies in enhancing mental health preparedness and response during epidemics was compiled from the perspectives of individuals with experience in participating in this domain (Fig. 1).

### Policy-making, planning, and preparedness strategies

The present study delved into policy-making, planning, and preparedness strategies for mental health, underscoring their critical importance prior to major epidemics to ensure readiness for an effective response.

In this study, we emphasized that mental health should consistently be a top priority for national health-related organizations. Mental health governance is highlighted not only as a fundamental human right but also essential for societal well-being, given the significant global burden of mental health disorders [8]. Recommendations stress the need for strong leadership and governance in mental health, involving active participation from public health advocates and governments to develop comprehensive policies and response programs [6, 8]. Effective governance, planning, supervision, and accountability are crucial for achieving organizational goals [20]. The Iranian Constitution guarantees healthcare rights for all citizens, and the national mental health program, initiated in 1986 and expanded since, illustrates ongoing efforts to integrate mental health services nationwide [21]. Although attention has been paid to mental health strategies in Iran's national disaster preparedness programs in the recent years, it is necessary to pay attention to specific solutions in the pandemics and to implement them. It is suggested to develop a pandemic specific mental health preparedness plan based on the national disaster preparedness plan.

To address mental health preparedness and response during pandemics like COVID-19, participants stressed the importance of policymaking and legislation. Previous research has also emphasized the need for comprehensive policies and legal frameworks [22]. These include interventions such as national mental health support plans, increased political commitment, and strategies tailored to pandemic-induced mental health impacts [23, 24]. Furthermore, there is consensus on the necessity of prioritizing mental health in epidemic responses. This involves enhancing mental health services, research, education, and allocating more budgets [25]. For instance, McCartan et al. [26] highlighted the importance of policy responses for mental health improvement, stressing the need for further research [26]. However, challenges remain in effectively implementing mental health policies during pandemics. More research is necessary to assess policy efficacy and identify areas for improvement. Comparative analyses with studies from other regions can offer valuable insights into best practices and strategies for addressing these challenges.

In our study, planning emerged as a crucial strategy for effective response before major epidemics. Healthcare systems bear the responsibility of planning for emergency response [27, 28]. However, many lack comprehensive plans to enhance capacity and deliver healthcare services during emergencies [27]. This resonates with prior research emphasizing proactive planning for epidemics and disasters [29], alongside implementing prevention programs and advocating for tailored initiatives [6]. Integrating mental health interventions into public health preparedness and emergency response plans is essential for addressing epidemics effectively [15]. This study suggests that although mental health programs have been integrated into Iran's primary health care system, it is necessary to prepare the primary health care system to provide comprehensive mental health services in pandemics through a proactive approach.

This study showed that it is necessary to invest more in education and training to provide mental health services in pandemics. The study participants emphasized training and exercise as vital components of preparedness programs for mental health response during epidemics. This aligns with existing literature highlighting the significance of disaster preparedness in reducing community harm [30]. Training and exercise are recognized as essential aspects of disaster preparedness efforts [31]. Similarities exist between managing epidemics and other natural disasters, underscoring the importance of drafting emergency scenarios, implementing preventive measures, and conducting training and drills. Furthermore, engaging efficient human resources, promoting public participation, and employing Community-Based Disaster Risk Management (CBDRM) principles are critical [27]. The World Health Organization (WHO) advocates for prioritizing healthcare professional training for disaster response as a national and local priority, irrespective of a country's disaster experience [32]. Incorporating real-life scenarios into training programs is also essential for effective education and learning [33]. Our findings resonate with previous studies, emphasizing the importance of training and exercise in disaster preparedness for effective mental health response during epidemics.

### Response strategies

Another noteworthy discovery in our study pertains to the strategies associated with the response phase of mental health during epidemics.

The results showed that although in Iran the united commanding of health is under the responsibility of the Ministry of Health, in practice, various organizations operate without coordination with national policies and programs. Implementing different programs with different approaches will not only not help people, but will cause them more confusion and anxiety. Participants highlighted the necessity of a unified and centralized command structure during epidemic response. This initial response phase necessitates the establishment of a robust command and control system to minimize uncertainty by efficiently processing information and mitigating unknown variables [34]. The response strategies adopted by different countries or regions are significantly influenced by their priorities and contextual conditions [27]. Notably, the implementation of an incident command system (ICS) has proven effective in managing the COVID-19 pandemic, facilitating improved communication, resource allocation, and overall safety measures [35]. The ICS framework aims to streamline command and control operations for swift and effective disaster response. Cook [36] demonstrated the efficacy of ICS implementation during the COVID-19 crisis, emphasizing its ability to mobilize personnel, assess situations, and develop comprehensive response plans. Despite its limitations, ICS provides a structured approach for disaster planning and response [36]. The examination of previous experiences, particularly with COVID-19 in Iran, sheds light on the relevance and effectiveness of the incident command system (ICS) as proposed by our contributors [37]. Evaluating the applicability and efficiency of ICS in the current context is crucial, considering its potential role in managing future epidemics and disasters. Iran's experience in COVID-19 showed that the monitoring, supervision and accountability of judicial institutions can play an effective role in the implementation of the incident management system by the Ministry of Health.

The high volume of providing mental health services during the pandemic usually is beyond the capacity of the existing mental health human resources. This causes them to experience fatigue and premature burnout. Lack of problem solving skills and mental resilience in these people and emotional actions aggravate this problem. It means that during pandemics like COVID-19, effective management of human resources is crucial for mental health response strategies. Participants emphasized the importance of supporting responders, addressing their psychological and educational needs, and mobilizing adequate personnel during the response phase. Studies have highlighted the heightened stress, fatigue, and psychological distress among healthcare workers due to increased workloads and risks during the COVID-19 pandemic [25, 38]. Consequently, there is a strong emphasis on providing social and psychological support for frontline workers, including targeted psychosocial support programs [39]. Recommendations from a study by Jahanmehr and colleagues (2022) underscore the necessity of planning for psychological counseling and providing welfare facilities to alleviate psychological pressures on healthcare staff [40]. Training programs aimed at enhancing healthcare workers' mental health knowledge and skills are also essential [41]. Furthermore, strategies such as strengthening the mental health workforce, optimizing roles, and maximizing existing resources are critical components identified in the literature [22, 29]. This study suggests that it is necessary to mobilize other human resources for this action in addition to strengthening the mental health workers' abilities.

It is clear that for the implementation of a program, it is necessary to allocate funds and financial resources. In addition, insurance coverage of mental health services is one of the programs that should be considered. Participants in this study stressed the critical need for financial resources, emphasizing that allocating funds to cover mental health expenses is essential during epidemics. The World Health Organization suggests that countries respond by increasing budgets and enhancing personnel capacity for mental health services amid the COVID-19 pandemic, anticipating heightened pressure on national and international mental health services soon [11]. Molebatsi et al. [23] highlighted the importance of investing in psychological support services and integrating them into national healthcare systems [23]. Therefore, policymakers are urged to prioritize mental health services and research by paying attention to policies, budgets, and the allocation of financial resources, considering the long-term impact of pandemics on mental well-being [41]. Additionally, advocating for public budget support to ensure access to mental health treatments is recommended [15, 42]. It should be noted that not allocating enough funds to mental health programs will multiply short-term and long-term mental impacts caused by epidemics.

Another essential component of mental health response during epidemics is strengthening mental health infrastructure. While prioritizing the development of mental health infrastructure is critical, particularly in low- and middle-income countries [24], our study emphasizes the necessity of integrated healthcare systems. Such systems should effectively bridge the gap between physical and mental health, ensuring accessibility, cost-effectiveness, and seamless integration of mental health services into primary care [25, 39, 43]. As highlighted in previous research, there's a growing recognition of the importance of integrating mental health services into primary care settings to enhance their reach and reduce stigma [39]. It's crucial to acknowledge the establishment of a public response telephone system like the 4030 system during the COVID-19 pandemic in Iran. The Ministry of Health and Medical Education in Iran has launched a psychological assessment platform to provide additional support for mental health initiatives. Additionally, recognizing the role of non-governmental organizations (NGOs) and their contributions during emergencies in Iran is essential for understanding the comprehensive response to such crises.

The results of this study emphasized that in addition to the surveillance systems for infectious diseases, health systems need to establish mental health surveillance systems. The importance of monitoring mental health before, during, and after epidemics was underscored by participants as a key response strategy. Evaluation and monitoring of the mental/psychiatric conditions of affected populations should be part of the intervention in the early stages of a pandemic and extend beyond, incorporating programs to monitor mental health for sufficient responsiveness to anticipated mental health issues [10]. Additionally, supporting the continuity and sustainability of monitoring after the acute phase of an epidemic is crucial, as mental health issues may persist or emerge later [25, 44]. While our study highlights the significance of this aspect, it's essential to consider practical solutions for monitoring and supervision, especially in the context of Iran. Drawing from global experiences, successful initiatives such as those observed in European countries, where electronic health records (EHR) were utilized to track mental health trends during the COVID-19 pandemic, offer valuable insights. These initiatives involved collecting data on new episodes of depression or anxiety, prescription patterns, and healthcare visits related to mental health issues [45]. Implementing similar mechanisms in Iran could provide a robust framework for monitoring and addressing mental health concerns during and after epidemics. Additionally, integrating pre-pandemic data with longitudinal follow-up assessments, as demonstrated in other studies, can offer unique insights into vulnerabilities and inform targeted interventions [46]. By leveraging international experiences and adapting successful strategies to the Iranian context, we can enhance our capacity for monitoring and addressing mental health needs throughout epidemic situations.

Research complements monitoring efforts by providing valuable insights into mental health trends and responses during epidemics. It serves as a crucial tool for understanding the effectiveness of monitoring strategies and informing targeted interventions. Participants underscored the critical need for attention to research, emphasizing the importance of increasing investment in mental health research. This investment can generate evidence to guide the development and implementation of effective mental health policies and programs [23]. Furthermore, conducting studies in various subgroups and prospective studies to assess changes over time can deepen our understanding of social and psychological responses. Additionally, examining the impact of social media and past experiences regarding disease outbreaks can provide valuable insights into developing more targeted interventions [47, 48].

The participants emphasized the importance of the participation of various sectors and coordination among them as strategies during the response phase of epidemics. In the study by Molebatsi et al. [23], enhancing the collaboration and participation among different sectors and stakeholders involved in mental health was highlighted to ensure a comprehensive and coordinated response to the mental health needs of individuals and communities affected during the COVID-19 pandemic [23]. Beckstein et al. [49] also emphasized collaborative efforts. The results of this study highlighted the importance of collaboration among mental health professionals, healthcare providers, policymakers, and social organizations for developing comprehensive strategies and programs to address mental health needs during the outbreaks of communicable diseases [49]. In this regard, the comprehensive approach implemented in China for an effective mental health response, which is coordinated and facilitated through various systems, including government, academic societies, universities, hospitals, and non-profit organizations, is noteworthy [42].

Identification and attention to vulnerable groups in terms of mental health constitute another response strategy during epidemics. Various studies have emphasized supporting vulnerable groups [23, 24, 41, 49]. Since specific populations may be disproportionately affected by the mental health impacts of epidemics, targeted interventions for vulnerable populations, including children, adolescents, older adults, individuals with pre-existing mental health conditions, and marginalized communities, are necessary [39]. Efforts to provide targeted mental health support in vulnerable populations may include ensuring access to mental health services, addressing social determinants of mental health, promoting equity in healthcare [43], identifying individuals susceptible to mental disorders, and implementing measures to maintain and improve their mental health [50].

Many studies have shown that it is not possible to improve mental health without education, inter-sectorial coordination and public participation. Based on this, investing in community-oriented measures and facilitating Community Based Organizations, and other community groups for mental health interventions should be given serious attention by health managers and policy makers. It is crucial to strengthen community-based mental health services to ensure accessibility and responsiveness to the needs of individuals and communities [23, 24, 51]. Mental health services should be community-based, evidence-based, accessible, fair, and proportionate to the existing mental health capacity [6]. Various studies have emphasized the importance of psychological first aid and the provision of necessary educational programs in this regard [25]. Implementing routine protocols for screening and assessing mental health, early intervention, and appropriate referral for treatment [39], raising awareness among the general population and healthcare providers about clinical manifestations of the disease for early diagnosis [47], establishing multidisciplinary mental health teams, creating safe counseling services with better access for disadvantaged individuals, and implementing mechanisms for monitoring, reporting, and intervening in suicides have also been mentioned [10]. Furthermore, the need to maintain the continuity of mental health services is emphasized, and access to psychological assistance should be available whenever needed, with sensitivity to specific arrangements related to the pandemic [47].

During epidemics, effective communication and information management regarding mental health are crucial. Providing accurate information from credible sources helps reassure the public and prevents the spread of rumors [50]. Governments and health authorities should promptly address misinformation, ensuring public security and psychological well-being [52]. Access to up-to-date information about the disease spread is vital as emphasized by Chew QH and colleagues (2020) [47]. Strategies such as limiting news consumption, avoiding misinformation, and relying on credible sources are recommended [9]. Establishing a robust health information management system is necessary for monitoring mental health care in communities [15]. Furthermore, researchers can leverage artificial intelligence for predictive models and community-based interventions, focusing on developing innovative digital solutions for information systems to enhance mental health communication during crises [6].

Public education and awareness-building about mental health are crucial during epidemics [22, 24, 25, 41, 53]. Initiatives include anti-stigma awareness programs, educational campaigns, and self-care strategies to reduce stigma and promote coping techniques [39, 47]. Recommendations encompass promoting positive behaviors, engaging in physical activities, and seeking professional help when needed [2, 54].

Technology and telemedicine play a vital role in mental health response, with a focus on digital interventions and remote services [24, 29, 55]. Teletherapy and online mental health services offer effective alternatives to in-person treatment, ensuring continuous access to support during quarantine [25]. Decision-makers should prioritize the development of digital strategies for mental health care, considering factors like social inequalities and digital divides. Telemedicine and digital psychiatry hold promise for future disaster response, but improvements are necessary [10].

Social support is a vital aspect of mental health response during epidemics [39, 41, 47, 54, 56] Community-centered programs and initiatives, including virtual support groups, online forums, and helplines, enhance social connections and reduce isolation [39, 41]. Utilizing technology and social media facilitates communication and fosters optimism for coping with the epidemic [47]. Clear communication between authorities and the public is crucial at the policy level of healthcare [54]. Building public trust horizontally among people and vertically between the public and their institutions is recommended [56]. Additionally, emphasis is placed on building resilience through community participation and social psychological support [29]. Community resilience is essential for pandemic preparedness [7] and strengthening the healthcare system [50].

Economic support is crucial in the mental health response to epidemics, addressing socio-economic inequalities and supporting vulnerable populations [15, 57]. McGrath et al. [57] highlighted the importance of mitigating financial hardships resulting from the COVID-19 pandemic to improve mental health outcomes. Social determinants of health, such as economic status, can be modified through community-focused interventions, including services like debt advice, food insecurity interventions, and active labor market programs [57]. Maulik et al. [6] also emphasized the need for support from civil societies and employers to cope with the increasing mental pressure [6].

Considering the qualitative nature of our study, it is essential to acknowledge several limitations. Firstly, due to challenges in accessing all individuals involved in mental health during the crisis, our sample size was small, which limits the generalizability of our findings. Additionally, conducting some interviews virtually may have impacted the depth of data collected compared to face-to-face interactions. Moreover, the participants were predominantly from psychology and psychiatry backgrounds, potentially leading to a skewed perspective and overlooking viewpoints from other stakeholders, such as the broader community. Including diverse perspectives could have enriched our study and provided more comprehensive insights. These limitations underscore the importance of replicating our findings in different settings to validate the proposed strategies and enhance mental health preparedness for future crises and community well-being.

## Conclusion

Effective management of epidemics necessitates the implementation of tailored mental health responses. This requires a concerted effort from policymakers, managers, and decision-makers within the mental health domain to prioritize comprehensive planning, education, research, and the development of technological infrastructure. Drawing from the invaluable lessons learned during the COVID-19 pandemic, it is crucial to guide the implementation of training programs, guidelines, and resource allocation based on these experiences. The insights and experiences of managers and experts in mental health, rooted in their expertise and knowledge, have significantly influenced the conclusions of this study. These contributions enrich the healthcare system with invaluable resources, empowering us to enhance epidemic response strategies significantly.

### Supplementary Information

Supplementary Material 1.

## Data Availability

The datasets used and/or analysed during the current study are available from the corresponding author on reasonable request.

## References

[1] Thangaswamy GC, Arulappan J, Anumanthan S, Jayapal SK. Trends and determinants of mental health during COVID-19 pandemic: implications and strategies to overcome the mental health issues-a rapid review from 2019–2020. Int J Nutr pharmacol Neurol Dis. 2021;11(1):1–6. 10.4103/ijnpnd.ijnpnd_86_20

[2] Sharma H, Verma S. Preservation of physical and mental health amid COVID-19 pandemic: recommendations from the existing evidence of disease outbreaks. Int J Acad Med. 2020;6(2):76. 10.4103/IJAM.IJAM_47_20

[3] Cénat JM, Felix N, Blais-Rochette C, Rousseau C, Bukaka J, Derivois D, et al. Prevalence of mental health problems in populations affected by the Ebola virus disease: a systematic review and meta-analysis. Psychiatry Res. 2020;289:113033. 32388176 10.1016/j.psychres.2020.113033

[4] Santomauro DF, Herrera AMM, Shadid J, Zheng P, Ashbaugh C, Pigott DM, et al. Global prevalence and burden of depressive and anxiety disorders in 204 countries and territories in 2020 due to the COVID-19 pandemic. Lancet. 2021;398(10312):1700–12. 10.1016/S0140-6736(21)02143-7 PMC8500697 34634250

[5] Kola L, Kumar M, Kohrt BA, Fatodu T, Olayemi BA, Adefolarin AO. Strengthening public mental health during and after the acute phase of the COVID-19 pandemic. Lancet. 2022;399(10338):1851–2. 10.1016/S0140-6736(22)00523-2 PMC8947778 35339230

[6] Maulik PK, Thornicroft G, Saxena S. Roadmap to strengthen global mental health systems to tackle the impact of the COVID-19 pandemic. Int J Ment Heal Syst. 2020;14:1–13. 10.1186/s13033-020-00393-4 PMC7389161 32742305

[7] Lindert J, Jakubauskiene M, Bilsen J. The COVID-19 disaster and mental health—assessing, responding and recovering. European J Public Health. 2021;31(Supplement_4):iv31–5. 34751367 10.1093/eurpub/ckab153 PMC8576295

[8] Radfar A, Ferreira MM, Sosa JP, Filip I. Emergent crisis of COVID-19 pandemic: mental health challenges and opportunities. Front Psychiatry. 2021;12:631008. 10.3389/fpsyt.2021.631008 PMC8326372 34349675

[9] Samantaray NN, Pattanaik R, Srivastava K, Singh P. Psychological management of mental health concerns related to COVID-19: A review of guidelines and recommendations. Ind Psychiatry J. 2020;29(1):12. 33776270 10.4103/ipj.ipj_81_20 PMC7989461

[10] Talevi D, Pacitti F, Socci V, Renzi G, Alessandrini MC, Trebbi E, et al. The COVID-19 outbreak: impact on mental health and intervention strategies. J Psychopathol. 2020;26(2):162–8.

[11] Brunier A, Drysdale C. COVID-19 disrupting mental health services in most countries, WHO survey. World Health Organization. 2020. https://www.who.int/news/item/05-10-2020-covid-19-disrupting-mental-health-services-in-most-countries-who-survey. Accessed 3 Mar 2023.

[12] Zhang J, Wu W, Zhao X, Zhang W. Recommended psychological crisis intervention response to the 2019 novel coronavirus pneumonia outbreak in China: a model of West China Hospital. Precision Clin Med. 2020;3(1):3–8. 35960676 10.1093/pcmedi/pbaa006 PMC7107095

[13] Irandoost SF, Yoosefi Lebni J, Safari H, Khorami F, Ahmadi S, Soofizad G, et al. Explaining the challenges and adaptation strategies of nurses in caring for patients with COVID-19: a qualitative study in Iran. BMC Nurs. 2022;21(1):170. 35765051 10.1186/s12912-022-00937-8 PMC9238071

[14] Raesi A, Hajebi A, Rasoulian M, Abbasinejad M. The effects of COVID-19 on mental health of the society: a dynamic approach in Iran. Med J Islam Repub Iran. 2020;34:102. 33316003 10.34171/mjiri.34.102 PMC7722961

[15] Otu A, Charles CH, Yaya S. Mental health and psychosocial well-being during the COVID-19 pandemic: The invisible elephant in the room. Int J Ment Heal Syst. 2020;14(1):38. 10.1186/s13033-020-00371-w PMC7257210 32514302

[16] Clemente-Suárez V, Navarro-Jiménez E, Jimenez M, Hormeño-Holgado A, Martinez-Gonzalez M, Benitez-Agudelo J. Impact of COVID-19 pandemic in public mental health: an extensive narrative review. Sustainability. 2021;13(6):3221. 10.3390/su13063221

[17] Elo S, Kyngäs H. The qualitative content analysis process. J Adv Nurs. 2008;62(1):107–15. 18352969 10.1111/j.1365-2648.2007.04569.x

[18] Graneheim UH, Lundman B. Qualitative content analysis in nursing research: concepts, procedures and measures to achieve trustworthiness. Nurse Educ Today. 2004;24(2):105–12. 14769454 10.1016/j.nedt.2003.10.001

[19] Lincoln YS, Guba EG. But is it rigorous? Trustworthiness and authenticity in naturalistic evaluation. New Dir Program Eval. 1986;1986:73–84. 10.1002/ev.1427

[20] Mosadeghrad A. Essentials of healthcare organization and management. Tehran: Dibagran Tehran; 2015. p. 17.

[21] Yasamy M, Shahmohammadi D, Bagheri Yazdi S, Layeghi H, Bolhari J, Razzaghi E, et al. Mental health in the Islamic Republic of Iran: achievements and areas of need. EMHJ-East Mediterr Health J. 2001;7(3):381–91. 10.26719/2001.7.3.381 12690757

[22] Javed A, Lee C, Zakaria H, Buenaventura RD, Cetkovich-Bakmas M, Duailibi K, et al. Reducing the stigma of mental health disorders with a focus on low-and middle-income countries. Asian J Psychiatr. 2021;58: 102601. 33611083 10.1016/j.ajp.2021.102601

[23] Molebatsi K, Musindo O, Ntlantsana V, Wambua GN. Mental health and psychosocial support during COVID-19: a review of health guidelines in sub-Saharan Africa. Front Psych. 2021;12:571342. 10.3389/fpsyt.2021.571342 PMC8172960 34093251

[24] Alshammari MA, Alshammari TK. COVID-19: A new challenge for mental health and policymaking recommendations. J Infect Public Health. 2021;14(8):1065–8. 34174536 10.1016/j.jiph.2021.05.020 PMC8188780

[25] Chaudhury P, Banerjee D. RETRACTED:“Recovering With Nature”: A Review of Ecotherapy and Implications for the COVID-19 Pandemic. Front Public Health. 2020;8:604440. 10.3389/fpubh.2020.604440 PMC7758313 33363096

[26] McCartan C, Adell T, Cameron J, Davidson G, Knifton L, McDaid S, et al. A scoping review of international policy responses to mental health recovery during the COVID-19 pandemic. Health Research Policy and Systems. 2021;19:1–7. 33823855 10.1186/s12961-020-00652-3 PMC8022299

[27] Yari A, Motlagh ME, Zarezadeh Y. COVID-19: 12 Tips for Crisis Management. Health Emerg Disasters Q. 2022;7(2):59–62. 10.32598/hdq.7.2.396.1

[28] Richmond JG, Tochkin J, Hertelendy AJ. Canadian health emergency management professionals’ perspectives on the prevalence and effectiveness of disaster preparedness activities in response to COVID-19. Int J Disaster Risk Reduction. 2021;60:102325. 36570631 10.1016/j.ijdrr.2021.102325 PMC9764162

[29] Roy A, Singh AK, Mishra S, Chinnadurai A, Mitra A, Bakshi O. Mental health implications of COVID-19 pandemic and its response in India. Int J Soc Psychiatry. 2021;67(5):587–600. 32873106 10.1177/0020764020950769 PMC7468668

[30] Bhattacharya S, Singh A, Semwal J, Marzo RR, Sharma N, Goyal M, et al. Impact of a training program on disaster preparedness among paramedic students of a tertiary care hospital of North India: A single-group, before-after intervention study. J Educ Health Promot. 2020;9:5. 32154300 10.4103/jehp.jehp_423_19 PMC7032020

[31] Yari A, Zarezadeh Y, Fatemi F, Ardalan A, Vahedi S, Yousefi-Khoshsabeghe H, et al. Disaster safety assessment of primary healthcare facilities: a cross-sectional study in Kurdistan province of Iran. BMC Emerg Med. 2021;21:1–9. 33622259 10.1186/s12873-021-00417-3 PMC7903750

[32] Achora S, Kamanyire JK. Disaster preparedness: Need for inclusion in undergraduate nursing education. Sultan Qaboos Univ Med J. 2016;16(1):e15. 26909207 10.18295/squmj.2016.16.01.004 PMC4746037

[33] Yang YN, Xiao L, Cheng HY, Zhu JC, Arbon P. Chinese nurses’ experience in the Wenchuan earthquake relief. Int Nurs Rev. 2010;57(2):217–23. 20579157 10.1111/j.1466-7657.2009.00795.x

[34] Chaudhury KS, Nibedita A, Mishra PK. Command and control in disaster management. Int J Compu Sci Issues (IJCSI). 2012;9(4):256.

[35] Farcas A, Ko J, Chan J, Malik S, Nono L, Chiampas G. Use of incident command system for disaster preparedness: a model for an emergency department COVID-19 response. Disaster Med Public Health Prep. 2021;15(3):e31–6. 32576330 10.1017/dmp.2020.210 PMC7371845

[36] Cook J. Incident command in the time of COVID-19. Lab Med. 2020;51(6):e78–82. 32909043 10.1093/labmed/lmaa073 PMC7499792

[37] Yari A, Yousefi Khoshsabegheh H, Zarezadeh Y, Amraei M, Soufi Boubakran M, Motlagh ME. Iranian primary healthcare system’s response to the COVID-19 pandemic using the healthcare incident command system. PLoS ONE. 2023;18(8):e0290273. 37607162 10.1371/journal.pone.0290273 PMC10443878

[38] Gholamzad S, Heydari Yazdi AS, Salimi Z, Saeidi N, Hajebi Khaniki S, Noori R, et al. Resilience and coronavirus anxiety in Iran: Online survey among healthcare workers and non-healthcare workers. J Fundamentals Mental Health. 2023;25(5):297–302.

[39] Tausch A, e Souza RO, Viciana CM, Cayetano C, Barbosa J, Hennis AJM. Strengthening mental health responses to COVID-19 in the Americas: A health policy analysis and recommendations. Lancet Regional Health - Americas. 2022;5:100118. 10.1016/j.lana.2021.100118 PMC8782269 35098200

[40] Jahanmehr N, Siamiaghdam A, Daneshkohan A. Covid-19 in Iran: a qualitative study of the experiences of health care workers. J School Public Health Institute Public Health Res. 2022;20(1):97–110.

[41] Kumar R, Singh A, Mishra R, Saraswati U, Bhalla J, Pagali S. A Review Study on the Trends of Psychological Challenges, Coping Ways, and Public Support During the COVID-19 Pandemic in the Vulnerable Populations in the United States. Front Psych. 2022;13:920581. 10.3389/fpsyt.2022.920581 PMC9300847 35873246

[42] Miu A, Cao H, Zhang B, Zhang H. Review of mental health response to COVID-19, China. Emerg Infect Dis. 2020;26(10):2482. 32620177 10.3201/eid2610.201113 PMC7510735

[43] Campion J, Javed A, Lund C, Sartorius N, Saxena S, Marmot M, et al. Public mental health: required actions to address implementation failure in the context of COVID-19. Lancet Psychiatry. 2022;9(2):169–82. 35065723 10.1016/S2215-0366(21)00199-1 PMC8776278

[44] Madani SMS, Bahramnejad A, Farsi Z, Alizadeh A, Rajai N, Azizi M. Effectiveness of Psychological First Aid E-learning on the Competence and Empathy of Nurses in Disasters: A Randomized Controlled Trial. Disaster Med Public Health Prep. 2023;17:e420. 37357997 10.1017/dmp.2023.81

[45] Rodríguez-Blázquez C, Aldridge S, Bernal-Delgado E, Dolanski-Aghamanoukjan L, Estupiñán-Romero F, Garriga C, et al. Monitoring COVID-19 related changes in population mental health. Eur J Public Health. 2022;32(Supplement_3):ckac129. 276. 10.1093/eurpub/ckac129.276

[46] Kujawa A. Performance monitoring and mental health during the COVID-19 pandemic: Clarifying pathways to internalizing psychopathology. Biol Psychiatry Glob Open Sci. 2021;1(4):249–51. 34927125 10.1016/j.bpsgos.2021.10.001 PMC8671874

[47] Chew QH, Wei KC, Vasoo S, Chua HC, Sim K. Narrative synthesis of psychological and coping responses towards emerging infectious disease outbreaks in the general population: practical considerations for the COVID-19 pandemic. Singapore Med J. 2020;61(7):350. 32241071 10.11622/smedj.2020046 PMC7926608

[48] Azizi M, Bidaki R, Ebadi A, Ostadtaghizadeh A, Tafti AD, Hajebi A, et al. Psychological distress management in iranian emergency prehospital providers: a qualitative study. J Educ Health Promot. 2021;10(1):442. 35071648 10.4103/jehp.jehp_351_21 PMC8719565

[49] Beckstein A, Chollier M, Kaur S, Ghimire AR. Mental wellbeing and boosting resilience to mitigate the adverse consequences of the COVID-19 pandemic: A critical narrative review. SAGE Open. 2022;12(2):21582440221100456. 10.1177/21582440221100455

[50] Shrivastava SR, Shrivastava PS. COVID-19 and impairment of mental health: public health perspective. Afr Health Sci. 2021;21(4):1527–32. 35283979 10.4314/ahs.v21i4.5 PMC8889819

[51] Alizadeh A, Khankeh HR, Barati M, Ahmadi Y, Hadian A, Azizi M. Psychological distress among Iranian health-care providers exposed to coronavirus disease 2019 (COVID-19): a qualitative study. BMC Psychiatry. 2020;20:1–10. 33028290 10.1186/s12888-020-02889-2 PMC7538532

[52] Salari N, Hosseinian-Far A, Jalali R, Vaisi-Raygani A, Rasoulpoor S, Mohammadi M, et al. Prevalence of stress, anxiety, depression among the general population during the COVID-19 pandemic: a systematic review and meta-analysis. Glob Health. 2020;16(1):1–11. 10.1186/s12992-020-00589-w PMC7338126 32631403

[53] Baldaçara L, Da Silva AG, Pereira LA, Malloy-Diniz L, Tung TC. The management of psychiatric emergencies in situations of public calamity. Front Psych. 2021;12:15. 10.3389/fpsyt.2021.556792 PMC7905390 33643085

[54] Tsamakis K, Tsiptsios D, Ouranidis A, Mueller C, Schizas D, Terniotis C, et al. COVID-19 and its consequences on mental health. Exp Ther Med. 2021;21(3):1. 33603852 10.3892/etm.2021.9675 PMC7851613

[55] Khaleghi A, Mohammadi MR, Jahromi GP, Zarafshan H. New ways to manage pandemics: using technologies in the era of COVID-19: a narrative review. Iran J Psychiatry. 2020;15(3):236. 33193772 10.18502/ijps.v15i3.3816 PMC7603586

[56] Jakovljevic M, Bjedov S, Mustac F, Jakovljevic I. COVID-19 infodemic and public trust from the perspective of public and global mental health. Psychiatr Danub. 2020;32(3–4):449–57. 33370752 10.24869/psyd.2020.449

[57] McGrath M, Duncan F, Dotsikas K, Baskin C, Crosby L, Gnani S, et al. Effectiveness of community interventions for protecting and promoting the mental health of working-age adults experiencing financial uncertainty: a systematic review. J Epidemiol Community Health. 2021;75(7):665–73. 33931550 10.1136/jech-2020-215574 PMC8223661

