# Supplementary material for: Mental health preparedness and response to epidemics focusing on COVID-19 pandemic: a qualitative study in Iran
Source: BMC Public Health. 2024 Jul 24;24:1980. doi: 10.1186/s12889-024-19526-2 (PMC11270955; doi:10.1186/s12889-024-19526-2)
Supplement: Supplementary file 1 — Supplementary Material 1. [file 12889_2024_19526_MOESM1_ESM.docx]

**Qualitative Interview Guideline in Mental Health Preparedness and Response to Epidemics Focusing on COVID-19 Pandemic: A Qualitative Study in Iran**

**Introduction:**

Begin by introducing yourself and your role in this study.

Explain the purpose of the interview and ensure confidentiality.

**Questions:**

**Question 1:** How would you describe your positive and negative experiences in this field?

**Question 2:** In order to provide mental health response strategies, what issues and challenges have you encountered? Please describe them.

**Question 3:** What solutions do you employ to deal with these challenges?

**Question 4:** Based on your experiences during the COVID-19 crisis, describe the actions that the healthcare system should take in response to mental health issues during epidemics.
